# Effect of myofunctional therapy on snoring in obese patients: a randomized trial

**DOI:** 10.5935/1984-0063.20220073

**Published:** 2022

**Authors:** Thiare Sperger, Allan Cezar Faria Araujo, Carolina Ferraz de Paula Soares

**Affiliations:** 1 Universidade Estadual do Oeste do Paraná, Biosciences and Health - Cascavel - PR - Brazil; 2 Universidade Estadual do Oeste do Paraná, Biociences and Health, University Hospital of Western Paraná (Huop) - Cascavel - Paraná - Brazil; 3 Universidade São Paulo HCFMUSP, Otolaryngology - São Paulo - Brazil

**Keywords:** Myofunctional Therapy, Snoring, Obesity, Randomized Controlled Trial, Smartphone, Mobile Applications

## Abstract

**Objective:**

To analyze the effectiveness of myofunctional therapy (MT) in the treatment of habitual snoring in obese patients.

**Material and Methods:**

This randomized clinical trial consisted of an experimental group (n=14) that underwent MT and a control group (n=26) that performed nonspecific exercises for the treatment of snoring. The Epworth sleepiness scale (ESS), Pittsburgh sleep quality index (PSQI), and short-form health survey (SF-36) were applied before and after treatment. Snoring was assessed subjectively by asking the partner about improvement after treatment. The SnoreLab app was used for objective assessment.

**Results:**

There was no significant effect of MT on any of the SnoreLab variables analyzed when groups, time points or covariates (adherence, age, body mass index [BMI], neck circumference, and sex) were compared. Neck circumference (cm) and the Pittsburgh sleep quality index score were significantly higher after treatment. There was no change in the Epworth sleepiness scale score after treatment. A correlation was found between BMI and the Pittsburgh sleep quality index and between BMI and the functional capacity component of the SF-36. Patient adherence was similar between groups.

**Discussion:**

Apps for recording snoring are a useful tool to be explored. MT exerted no significant effect on habitual snoring in obese patients despite the reduction of the snore score in the experimental group. Therapy applied without exclusion criteria based on the severity of sleep breathing disorders and pharyngeal characteristics fails to achieve the results necessary to treat habitual snoring in obese patients.

## INTRODUCTION

The prevalence of overweight and obesity is increasing at an alarming rate in many countries^1^. In Brazil, the percentage of obese men and women in the adult population (≥18 years) has increased significantly between 2006-2019. There were 11.4% obese men in 2006 and this percentage increased to 19.5% in 2019. The percentage of obese women increased from 12.1% in 2006 to 21.0% in 2019^2^.

A higher body mass index (BMI) is known to be associated with daytime sleepiness, increased neck and waist circumferences, lower self-rated sleep quality, a higher risk of obstructive sleep apnea (OSA), lower sleep efficiency, and a higher snoring rate^3^. Regardless of the degree of sleep apnea, habitual snoring can be harmful even in the absence of other sleep disorders^4^. According to Rich et al. (2011)^5^, there is evidence that snoring is associated with a number of health problems, including sleepiness and metabolic syndrome. In a 10-year cohort study involving 0.5 million adults^6^, habitual snoring was associated with an increased risk of cardiovascular diseases, including ischemic heart disease and ischemic stroke. Sekizuka and Miyake (2020)^7^ reported that the longer the duration of snoring, the higher the incidence of hypertension, diabetes, and dyslipidemia. The authors also found that the prevalence of snoring increases with increasing age and BMI in men and women.

The differential diagnosis of simple snoring or OSA is made by polysomnography (PSG) or similar sleep studies. However, PSG as the gold standard method and polygraphs are costly and are not available in public health centers in Brazil. Another limitation of PSG is that it fails to diagnose the majority of patients with snoring and OSA^8^.

Regarding the assessment of snoring, in an epidemiological study, Rich et al. (2011)^5^ highlighted the fact that, although many studies have reported the effects of snoring, the condition is not measured routinely. The American Association of Sleep Medicine (AASM) recommends no gold standard method to measure snoring. The 2015 Manual for the Scoring of Sleep and Associated Events considers three different methods to be equivalent: acoustic sensors, nasal pressure transducers, and piezoelectric vibration sensors^9^. Within this context, smartphone apps designed to record snoring and to measure the nighttime frequency and snoring sound intensity for monitoring the effect of interventions may be valuable tools for patients who snore and for professionals who treat snoring^10^.

A nonsurgical treatment for primary snoring is myofunctional therapy (MT)^11^, which aims to improve posture, sensitivity, proprioception, tone, and mobility of the orofacial and pharyngeal muscles^12^. In a randomized controlled trial, Ieto et al. (2015)^13^ tested the effects of MT on snoring and observed a reduction in the bed partner’s perception of snoring, frequency of snoring, and total snore index.

Previous studies have shown that sleep-related breathing disorders are not diagnosed in more than one-third of patients with snoring and obesity who are on the waiting list for bariatric surgery, a fact delaying the treatment of these disorders^14^. Since patients with obesity and snoring are known to be at high risk for OSA and habitual snoring represents a health risk, compromising quality of life and increasing cardiovascular disease risk, the availability of a treatment based only on habitual snoring and BMI may be beneficial and effective.

Furthermore, the efficacy of MT in the treatment of snoring was confirmed in other populations that are not predominantly obese. Considering the difficulty of measuring snoring, with the need for a simpler and easily accessible method, this study aimed to analyze the effectiveness of MT in the treatment of snoring in obese patients using a smartphone app, subjective perception and quality of life before and after the MT.

## MATERIAL AND METHODS

This randomized clinical trial consisted of an experimental group (n=14) that underwent MT and a control group (n=26) that performed nonspecific oral exercises for the treatment of snoring. The participants were randomized 2:1 using an electronic system. The experimental group performed the exercises of the protocol proposed by Ieto et al. (2015)^13^ and the control group followed the protocol of Kayamori (2015)^15^.

Weekly therapy sessions were held for 3 months, totaling 12 sessions per patient. In addition to attending the therapy sessions, patients in both groups were instructed to perform the exercises at home three times a day, in which the patient should not go a day without performing the proposed exercises since treatment initiation.

The criteria for inclusion in the study were a minimum age of 18 years and maximum age of 65 years; BMI≥30kg/m^2^; possession of a smartphone that permits the use of the SnoreLab (Reviva Softworks Ltd., London, UK) and WhatsApp (Meta, Inc.) apps; agreement to sleep in separate rooms without the partner during the periods of sleep recording, and consent to participate in the study by signing the free informed consent form. Subjects who were unavailable for the treatment during the established period, those who were already undergoing any treatment for sleep disorders, users of benzodiazepines, continuous users of muscle relaxing agents, alcoholics, and subjects who did not attend four or more of the weekly MT sessions were excluded.

At the beginning and end of treatment, neck circumference, weight, and height were measured in both groups and the BMI was calculated. The patients of the experimental and control groups were asked to wash their nose with 0.9% saline solution before bedtime and to keep a diary for recording the adherence to the set of exercises prescribed three times a day.

As a subjective way of evaluating snoring, after the end of the treatment, the bedmates were asked: “Regarding your partner’s snoring, do you think it has improved after the treatment?” Just answer “yes” or “no”.

Snoring was evaluated objectively using the free version of the SnoreLab app developed for smartphones and available for the Android and IOS operating systems. This app records snoring, displays the sound intensity, and provides data for the analysis of snoring. The researcher listened to the audio recordings of all patients and those exhibiting external noise were excluded.

SnoreLab assigns volume ratings to the following four categories, permitting to view the time and percentage for each category: quiet; light: unlikely to disturb a bed partner - 40 to 50; loud: likely to disturb a bed partner - 51 to 60; epic: very likely to annoy a bed partner - over 61. In addition to the snore score, the SnoreLab app provides a measure of snoring intensity, where a higher score indicates louder or more frequent snoring, while a lower score indicates less intense or less frequent snoring.

The sum of the total night period recorded by the SnoreLab app was 10 days per patient. The first five recordings were obtained before the beginning of treatment and the other five recordings after the 12^th^ week, i.e., after completing the snoring treatment, in order to evaluate the outcome of MT in the experimental group. Thus, the means of the 5 days of assessment at each time point were extracted for each of the following parameters: snore score, snoring time (minutes), percent snoring time, and percentage of snoring intensity (quiet, light, loud, and epic).

The patients received the following instructions for the sleep recording periods: to sleep in a quiet and separate room from the bed partner; to place the device on the side of the bed, with the main microphone facing the patient; and to keep the device in the same place every night to ensure that the results of several nights could be compared.

The following questionnaires were applied before and after treatment: Epworth sleepiness scale (ESS), Pittsburgh sleep quality index (PSQI), and 36-item short-form health survey (SF-36).

To assess the effectiveness of MT in the treatment of snoring in obese patients, mixed/hierarchical linear models were constructed and fitted separately to the snoring indices: snore score, snoring time (minutes), percent snoring time, and percentage of snoring intensity [quiet (%), light (%), loud (%) and epic (%)]. The following predictor variables were considered (x-axis - fixed effect): group (experimental/control), time (pre/post), treatment adherence, neck circumference (cm), age (years), sex (male/female), and BMI, with the interaction between group and time, and adherence and patient as random intercept. Specifically for the percentage of epic snoring, the models were fitted assuming a Poisson probability distribution because a high proportion of the data were close or equal to zero. The R 3.6.0 software was used for the tests, in which the linear models with mixed effects were fitted using the ‘lmer’ function of the lme4 package. All graphs were constructed using the ggplot2 package. A chi-squared test was applied using the ‘chisq.test’ function of the stats package. A significance of α=0.05 was adopted for all tests.

The ethics committee on research involving humans, *Universidade Estadual do Oeste do Paraná*, approved the study (Approval number 3.832.391/2020). An amendment was approved during the period of data collection (approval number 4.243.037/2020), adapting the methodology during the COVID-19 pandemic. The study was registered in the Brazilian Clinical Trials Registry (ReBEC) (identifier: RBR-3h6nkn).

## RESULTS

**Figure 1:**
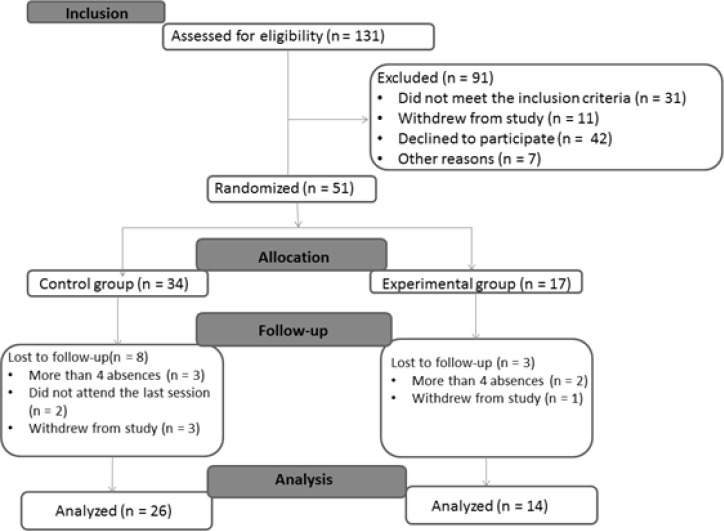
CONSORT flow diagram.

One hundred thirty-one patients were recruited and 91 were excluded, totaling 40 patients in the final analysis, with 26 patients in the control group and 14 in the experimental group (Figure 1).

**Figure 2:**
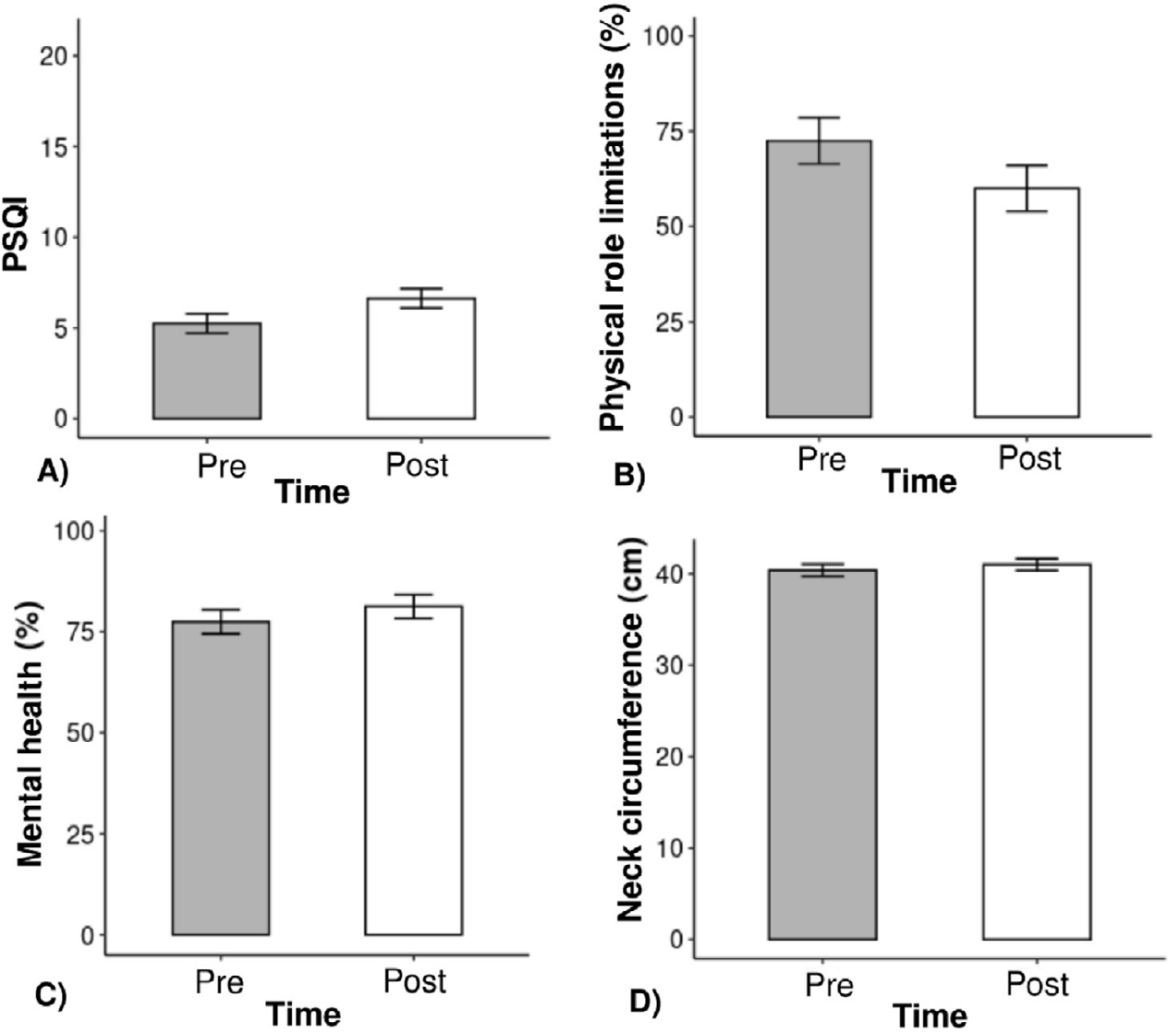
**A.** Difference in the Pittsburgh sleep quality index (PSQI) before and after treatment; **B.** SF-36: Difference in the percentage of role limitations due to physical health problems before and after treatment; **C.** SF-36: Difference in the percentage of emotional well-being before and after treatment; **D.** Difference in neck circumference (cm) before and after treatment.

The patients of the control and experimental groups had similar characteristics, enabling good inference from the models. Regarding the physical characteristics of the subjects, neck circumference (cm) was significantly greater after treatment (Table 1, Figure 2D).

**Table 1:** Demographic characteristics and treatment adherence.

Variable	Control group		Experimental group		p-value		
	Pre	Post	Pre	Post	Group^*^Time	Group	Time
**Demographic characteristics**							
BMI	37.26±6.08	37.3±5.61	34.86±3.83	35.2±3.99	0.469	0.203	0.467
NC	40.55±3.6	41.21±3.82	40.13±4.78	40.7±5.14	0.826	0.737	0.001^*^
Age	50.58±9.29		50.14±9.87		-	0.844	-
Sex	Female 14 [35%] Male 12 [30%]		Female 7 [17.5%] Male 7 [17.5%]		-	0.999	-
**Adherence**	195.46±49.80		189.57±71.77		-	0.762	-

Notes: BMI = Body mass index (kg/m^2^); NC = Neck circumference (cm); Group^*^Time = Indicates interaction.

The calculation of patient adherence based on the diary (Table 1) considered the number of sessions performed over the 12 weeks, in which 3 exercise sessions per day would be expected, totaling 270 sessions. Patient adherence was similar between the control and experimental groups (*p*=0.76), with some patients adhering better to treatment than others.

The global PSQI score was significantly higher after treatment (Table 2 and Figure 2A). Regarding the SF-36, the score for role limitations due to physical health problems was higher before treatment (Table 2 and Figure 2B), while the emotional well-being score was higher after treatment (Table 2 and Figure 2C).

**Table 2:** Pittsburgh sleep quality index, Epworth sleepiness scale, and SF-36 scores.

Variable	Control group		Experimental group		p-value		
	Pre	Post	Pre	Post	Group^*^Time	Group	Time
ESS	7.88±5.24	9.61±5.89	9.28±3.19	9.57±4.16	0.264	0.661	0.051
PSQI	5.23±2.98	6.96±3.68	5.28±3.53	6±3.55	0.311	0.659	0.006^*^
(SF-36)							
Physical functioning	75.19±23.22	71.73±23.02	72.5±24.94	75.71±18.9	0.235	0.927	0.672
RP	67.31±39.86	53.85±39.17	82.14±22.85	71.43±42.58	0.832	0.139	**0.049** ^*^
Pain	59.37±30.19	59.85±29.54	69.64±24.39	67.68±24.39	0.795	0.269	0.933
General health	70.85±19.94	67.96±22.36	70.71±25.93	72.86±23.51	0.392	0.730	0.687
Energy/fatigue	70.38±18.27	64.62±23.87	66.07±24.19	63.21±29.13	0.607	0.693	0.844
Social functioning	92.79±12.83	84.13±21.95	87.5±23	79.46±29.26	0.930	0.420	0.150
RE	85.9±30.07	74.36±35.67	71.44±36.65	71.43±43.08	0.327	0.402	0.183
Emotional well-being	82.92±16.02	78.31±18.35	78.29±21.72	76±21.4	0.526	0.564	0.035^*^

Notes: ESS = Epworth sleepiness scale; PSQI = Pittsburgh sleep quality index; SF-36 = Short-form health survey; RP = Role limitations due to physical health problems; RE = Role limitations due to emotional problems; Group^*^Time = Indicates interaction.

With respect to the effectiveness of MT in the treatment of snoring in obese patients evaluated using the SnoreLab app, there was no significant effect on any of the variables (snore score, snoring time, percent snoring time, or percentage of snoring intensity) between groups or between time points and covariates (adherence, age, BMI, neck circumference, and sex), indicating the lack of a significant effect of MT on snoring in obese patients (Table 3). Subjective assessment by asking the partner showed improvement which, however, was not significant.

**Table 3:** Snore indices (SnoreLab) and subjective assessment of snoring.

Variable	Control group		Experimental group			p-value		
	Pre	Post	Pre	Post		Group^*^Time	Group	Time
Snore indices (SnoreLab)^**^								
Score	52.99±35.01	54.96±35.30	57.38±50.97	54.21±50.29		0.616	0.935	0.968
Snoring time	148.11±57.78	150.76±57.65	137.49±77.49	144.03±80.29		0.510	0.828	0.660
Percent snoring time	41.05±16.28	41.06±16.59	34.88±17.33	35.70±16.97		0.846	0.371	0.869
Percentage of snoring intensity								
Quiet	57.67±18.34	58.54±17.06	62.45±20	64.64±16.97		0.819	0.351	0.600
Light	19.64±14.18	20.65±12.15	12.51±8.5	16.34±17.18		0.638	0.163	0.233
Loud	12.82±8.72	15.48±9.82	11.65±8.44	11.05±9.19		0.248	0.261	0.260
Epic	6.80±11.62	5.33±8.60	9.11±15.21	7.97±15.01		0.874	0.578	0.329
Partner questioning^***^	Yes 9 [25.71%] No 3 [8.51%]	-	Yes 20 [51.14%] No 3 [8.51%]	-		0.171	-	

Notes: Group^*^Time = Indicates interaction;

** Significance values obtained based on hierarchical regression models;

*** Three participants in the control group and 2 in the experimental group had no partner.

**Figure 3:**
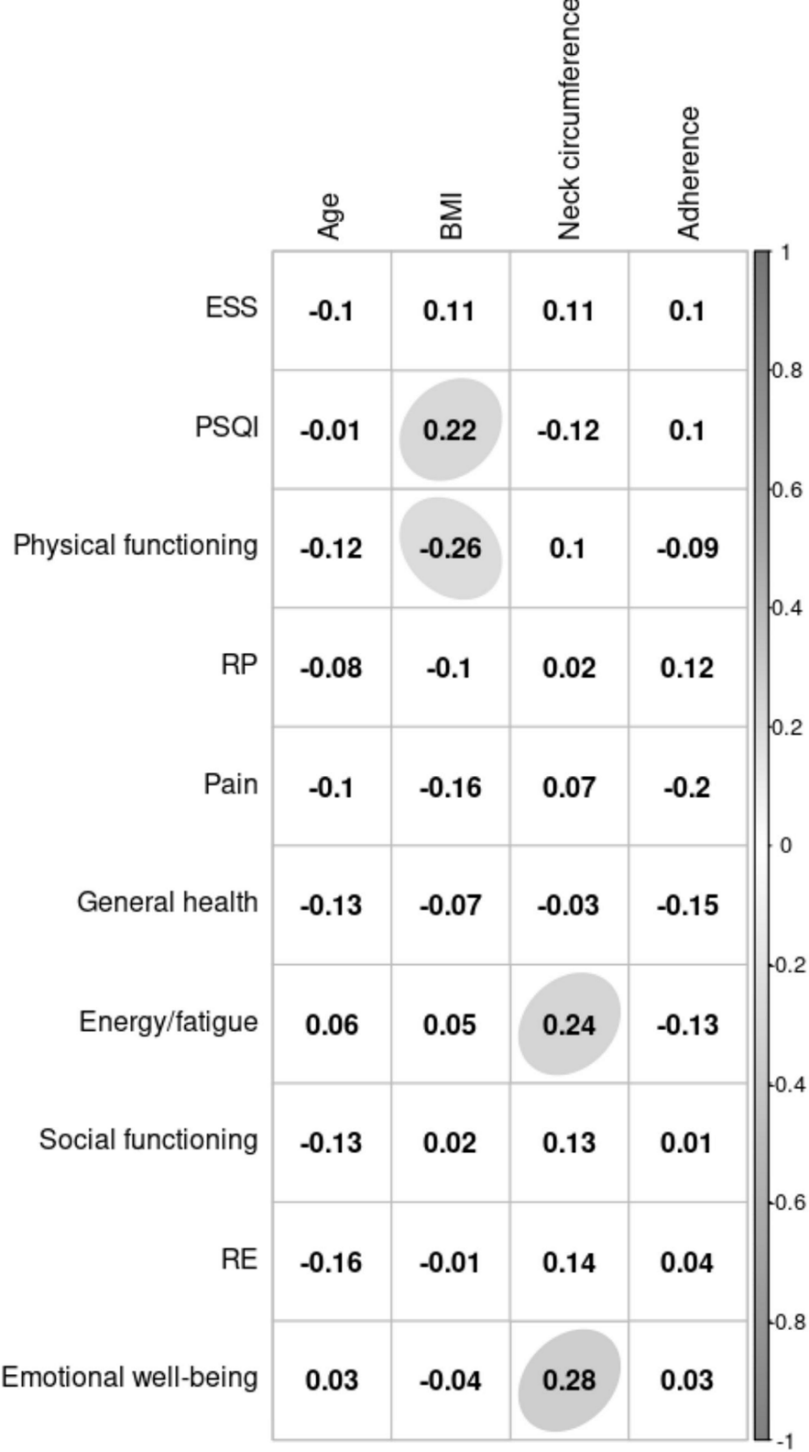
Pearson correlation matrix of sleep quality and quality of life parameters with the covariates age, BMI, neck circumference, and adherence rate. The ellipse indicates a significant *p*-value.

Additionally, a weak positive but significant correlation was observed between BMI and PSQI and a positive correlation between the functional capacity score of the SF-36 and BMI (Figure 3). As can also be seen in Figure 3, neck circumference showed a weak positive correlation with the energy/fatigue and emotional well-being components of the SF-36.

## DISCUSSION

This randomized study aimed to assess the effects of MT on habitual snoring in obese patients. Previous randomized studies^12,13,15-17^ reported lower mean BMI values than those observed in the present sample. Guimarães (2008)^12^ and Diaferia et al. (2013)^17^ studied the use of MT for the treatment of mild and moderate obstructive sleep apnea or independent of the degree of obstructive sleep apnea, respectively. Kayamori (2015)^15^ and Ieto et al. (2015)^13^ applied MT to treat primary snoring as well as obstructive sleep apnea.

No significant reduction in snoring was observed after 3 months and 12 MT sessions; although the snore score was lower in the experimental group. According to Baz et al. (2012)^18^, the selection of patients is crucial for a potential outcome of MT. We believe that the absence of oropharyngeal evaluation may have biased the results, probably because obstruction of the upper airway is caused not only by weakness and consequent collapse of the muscles but also by the volume of fat deposits around the tongue and pharynx in obese patients^19^ and other anatomical anomalies such as tonsil and tongue base hypertrophy, ankyloglossia, and macroglossia.

The increase of BMI in the experimental group after treatment, although not statistically significant, together with the lower adherence rate in this group when compared to the control group, raises the question regarding the influence of these factors on the efficacy of MT in the treatment of snoring in the population studied. There was a significant increase of neck circumference after treatment, in contrast to previous studies that reported a reduction of neck circumference in the MT group after treatment^13,15,17^. We may assume that this increase in neck circumference is a consequence of the increase in BMI found in both the experimental and the control group after the treatment period. We believe that the fact that the study took place during the SARS-CoV-2 pandemic may be linked to weight gain, as reported in some studies^20-22^.

Espinoza-López et al. (2021)^23^ suggested neck circumference as an anthropometric indicator since it is intimately related to different diseases, including obesity. Therefore, the correlation found between neck circumference and the energy/fatigue and emotional well-being health components of the SF-36 demonstrates that neck circumference is not only a physical measure but may also be an indicator of important health issues.

The methodology used to assess patient adherence was similar to that reported by Kayamori (2015)^15^. The author questioned the low adherence of patients, which may have prevented a decrease in the apnea and hypopnea index in that study. The same applies to the present study in which the low treatment adherence (70.21%) in the experimental group may have compromised the positive results of MT in reducing snoring in obese patients.

It is important to highlight that the detection of snoring currently depends on limited and ill-defined methods, both for recording and for analysis^24^. The objective assessment of snoring has become a topic of growing interest in an attempt to identify more accurate alternatives. Recently, a study comparing the accuracy of recorded snoring using the free version of the SnoreLab app to full-night polygraphic measurement showed good accuracy in measuring snoring >50% per night: 94.7% accuracy, 100% sensitivity, 94.1% specificity, positive predictive value of 66.6%, and negative predictive value of 100%. The SnoreLab app provided acceptable accuracy values for the measurement of snoring, with the percentage of total snoring assessed by SnoreLab being highly correlated with the proportion of snoring measured by polygraphy. The best agreement between the two methods was achieved when the sum of the loud and epic snoring rates obtained with the SnoreLab app was compared to the total snoring rate measured with the polygraph^25^.

The SnoreClock app used in the study by Chiang et al. (2021)^26^, as well as the SnoreLab of the present protocol, provides data on snoring duration and intensity, time of sleep recording, and snoring duration rate (%). Both apps possess a feature that allows users to focus on specific snoring events, facilitating the playback of the most noticeable snoring sounds. According to Camacho et al. (2015)^10^, the last feature is a valuable tool since it enables the user to view the graph of snoring sounds, to hear the sound, and to zoom in on the area of interest.

The bedmate’s perception of snoring revealed that the treatment improved the partner’s snoring, even in the control group. This finding confirms that the self-perception of snoring is inaccurate^27^ since no significant snoring reduction was obtained in the objective assessment.

Application of the ESS showed no change after treatment, unlike other studies^15,18,23^ that observed improvement in sleepiness. However, the present results agree with those reported by Ieto et al. (2015)^13^ and Kayamori (2015)^15^ who also found no improvement in sleepiness after MT.

The PSQI revealed poor sleep quality in the two groups and the results were worse after treatment, a finding that differs from previous studies^15,18^ showing improvement of sleep quality in the MT group after treatment. The worsening in the PSQI score after treatment may have been due to the time when patients completed the survey, which coincided with the period of the COVID-19 pandemic. During the sessions with the researcher, patients reported anxiety and concerns exacerbated by the situation of instability they were going through, which may have affected the quality of sleep.

A positive correlation was observed between BMI and the PSQI, in agreement with the study of Park et al. (2018)^28^ that found short sleep duration and poor sleep quality to be positively associated with obesity.

In agreement with the present results, studies^29,30^ have also demonstrated a correlation between BMI and functional capacity, a component of the SF-36, indicating that high anthropometric parameters of BMI are associated with unsatisfactory functional capacity. The role limitations due to physical health problems score was higher before treatment and there was an increase in the emotional well-being score after treatment. In contrast, Puhan et al. (2006)^16^ found no significant effect on any domain of the SF-36 after didgeridoo playing for the treatment of sleep disorders.

The present study aimed to evaluate the effectiveness of MT in the treatment of habitual snoring in obese individuals. Snoring was assessed objectively using the SnoreLab app and subjectively by asking the partner. There was no significant difference in objectively or subjectively assessed snoring between pre- and post-therapy, although a slight reduction in the snore score was observed in the experimental group. Considering the results, we believe that therapy applied without exclusion criteria based on the severity of sleep breathing disorders and pharyngeal characteristics fails to achieve the results necessary to treat habitual snoring in obese patients. Further studies are necessary to better assess the effect of MT on snoring in obese patients.
